# The association of long-term exposure to PM_2.5_ on all-cause mortality in the Nurses’ Health Study and the impact of measurement-error correction

**DOI:** 10.1186/s12940-015-0027-6

**Published:** 2015-05-01

**Authors:** Jaime E Hart, Xiaomei Liao, Biling Hong, Robin C Puett, Jeff D Yanosky, Helen Suh, Marianthi-Anna Kioumourtzoglou, Donna Spiegelman, Francine Laden

**Affiliations:** Channing Division of Network Medicine, Department of Medicine, Brigham and Women’s Hospital and Harvard Medical School, 401 Park Drive, Landmark Center, Boston, MA 02215 USA; Department of Environmental Health, Harvard T.H. Chan School of Public Health, 401 Park Drive, Landmark Center, Boston, MA 02215 USA; Department of Biostatistics, Harvard T. H. Chan School of Public Health, 665 Huntington Avenue, Boston, MA 02115 USA; Department of Epidemiology, Harvard T.H. Chan School of Public Health, 665 Huntington Avenue, Boston, MA 02115 USA; Maryland Institute for Applied Environmental Health, University of Maryland School of Public Health, 2234 School of Public Health, College Park, MD 20742 USA; Department of Public Health Sciences, Pennsylvania State University College of Medicine, 500 University Drive, Hershey, PA 17033 USA; Department of Health Sciences, Bouve College of Health Sciences, Northeastern University, 360 Huntington Avenue, Boston, MA 02115 USA

**Keywords:** PM_2.5_, Measurement error, Mortality, Air pollution

## Abstract

**Background:**

Long-term exposure to particulate matter less than 2.5 μm in diameter (PM_2.5_) has been consistently associated with risk of all-cause mortality. The methods used to assess exposure, such as area averages, nearest monitor values, land use regressions, and spatio-temporal models in these studies are subject to measurement error. However, to date, no study has attempted to incorporate adjustment for measurement error into a long-term study of the effects of air pollution on mortality.

**Methods:**

We followed 108,767 members of the Nurses’ Health Study (NHS) 2000–2006 and identified all deaths. Biennial mailed questionnaires provided a detailed residential address history and updated information on potential confounders. Time-varying average PM_2.5_ in the previous 12-months was assigned based on residential address and was predicted from either spatio-temporal prediction models or as concentrations measured at the nearest USEPA monitor. Information on the relationships of personal exposure to PM_2.5_ of ambient origin with spatio-temporal predicted and nearest monitor PM_2.5_ was available from five previous validation studies. Time-varying Cox proportional hazards models were used to estimate hazard ratios (HRs) and 95 percent confidence intervals (95%CI) for each 10 μg/m^3^ increase in PM_2.5_. Risk-set regression calibration was used to adjust estimates for measurement error.

**Results:**

Increasing exposure to PM_2.5_ was associated with an increased risk of mortality, and results were similar regardless of the method chosen for exposure assessment. Specifically, the multivariable adjusted HRs for each 10 μg/m^3^ increase in 12-month average PM_2.5_ from spatio-temporal prediction models were 1.13 (95%CI:1.05, 1.22) and 1.12 (95%CI:1.05, 1.21) for concentrations at the nearest EPA monitoring location. Adjustment for measurement error increased the magnitude of the HRs 4-10% and led to wider CIs (HR = 1.18; 95%CI: 1.02, 1.36 for each 10 μg/m^3^ increase in PM_2.5_ from the spatio-temporal models and HR = 1.22; 95%CI: 1.02, 1.45 from the nearest monitor estimates).

**Conclusions:**

These findings support the large body of literature on the adverse effects of PM_2.5_, and suggest that adjustment for measurement error be considered in future studies where possible.

## Background

Long-term exposures to ambient particulate air pollution have been associated with an increased risk of all-cause mortality in a number of studies [1-14]. In a recent meta-analysis of cohort studies [6], each 10 μg/m^3^ increase in particulate matter 2.5 μm or less in aerodynamic diameter (PM_2.5_) was associated with a 6.2% (95% confidence interval 4.1-8.4%) increased risk of all-cause mortality. As noted in that review, the assessment of exposure has varied across studies from city level measures to central ambient monitoring locations to complex land-use regression or other spatial exposure models, all of which have the potential to induce substantial measurement error. These measurement errors have been shown to be both classical, leading to attenuation of the exposure-response association, as well as Berkson, leading to increases in the width of the confidence intervals, resulting in overall biased results [15-19]. Each exposure modeling approach is likely subject to a different blend of classical and Berkson errors, however, to date, no studies have incorporated measurement error corrections into a study of the effects of air pollution on mortality.

In occupational and nutritional epidemiology, regression calibration is a widely used method to adjust for exposure measurement error [20,21]. This calibration is usually applied when surrogate exposure data have been collected in the majority of the participants, and measurements of the “true” exposure of interest (which themselves are also collected with error) have only been collected in a limited subset or are only available from an external study. Previous methods to incorporate calibration factors required that the exposures be time invariant; however, a more recent risk set regression calibration approach (RRC) now allows for the calibration of time-varying exposures in Cox proportional hazards models [22]. This new method makes it possible to utilize regression calibration in long-term studies of chronic exposure to air pollution.

In a previous analysis in a subset of members of the prospective Nurses’ Health Study living in the Northeastern and Midwestern US between 1992 and 2002, we observed increases in all-cause mortality, a multivariable adjusted hazard ratio (HR) of 1.26 (95% confidence interval: 1.02-1.54) for each 10 μg/m^3^ increase in 12-month average PM_2.5_ [2]. Our current objectives are to expand these analyses spatially to include the full nationwide cohort, incorporate additional follow-up, examine the impact of different methods of obtaining exposure estimates, and to apply RRC to adjust the obtained effect estimates for measurement error. We anticipated that PM_2.5_ exposures would be positively associated with all-cause mortality risk, that there would be differences in the magnitude and precision of the effect estimates from the two exposure modeling approaches, and that the magnitude of the effects would increase and the precision of the estimates would decrease after adjustment for exposure measurement error. To the best of our knowledge, this will be the first analyses to be able to adjust long-term PM_2.5_ and all-cause mortality effect estimates for measurement error, and will provide a framework for this method of adjustment for future studies.

## Methods

### Study population and assessment of outcome

The Nurses’ Health Study (NHS) is a long-term prospective cohort study of US female nurses. The cohort was initiated in 1976 when 121,700 married female US registered nurses, 30 to 55 years old, completed a mailed questionnaire and provided informed consent. At the study inception the nurses resided in eleven states; however, there is now at least one cohort member in all fifty states. Follow-up questionnaires, with response rates above 90%, are mailed every two years to update information on risk factors and the occurrence of major illnesses. The mailing lists for each questionnaire also provide updated information on residential address. Women were included in the current study if they were still alive in June of 2000 and had at least one geocoded address within the contiguous US between 2000 and 2006 A total of 108,767 women were available for analysis. We assessed incident cases of non-accidental mortality June 2000 through May 2006. Deaths were identified from state vital statistics records and the National Death Index or were reported by the families and the postal system and subsequently confirmed by death certificate.

### Exposure assessment

To ascertain each participant’s exposure to air pollution at each geocoded questionnaire mailing address, nationwide expansions of our spatio-temporal models of PM_2.5_ [23,24] were developed to estimate monthly PM_2.5_ exposures. These models and their previous use in assessing chronic PM exposures among the NHS cohort are described in detail elsewhere [3,25-29]. Briefly, a PM_2.3_ model was developed using monitor data from the US Environmental Protection Agency’s (USEPA) Air Quality System (AQS), the IMPROVE network, and Harvard research studies. The model also included meteorological and Geographic Information System (GIS)-derived covariates, such as: urban land use within 1 km, elevation, tract- and county-level population density, distance to nearest road for road classes A1-A3, and point-source emission density within 7.5 km. The model was evaluated using a cross-validation approach, where a sub-selection of monitors were held out to compare predicted to observed values [25-27] and were shown to exhibit little bias and high precision. For comparison to other studies that have used estimates from the nearest exposure monitoring location, we also calculated the monthly average PM_2.5_ from the nearest USEPA AQS monitoring location for all addresses 2000–2006.

### Exposure validation study

The details of the validation study and information on the populations included have been described previously [30-43]. Briefly, personal and ambient measurements of PM_2.5_ were available from a number of short-term panel exposure studies performed in nine US cities between 1999 and 2002. Personal exposures to PM_2.5_ of ambient origin (as the “true” exposures of interest) were estimated using the personal to ambient sulfate ratio, with ambient sulfate serving as a tracer for PM_2.5_ of ambient origin [44,45], or as the weighted average of indoor PM_2.5_ of ambient origin and ambient PM_2.5_, using home infiltration efficiencies and the proportion each subject spent indoors and outdoors [46]. Personal PM_2.5_ of ambient origin could only be calculated in five cities (Atlanta, GA, Baltimore, MD, Boston, MA, Seattle, WA and Steubenville, OH). Using the spatio-temporal model described above and data from the nearest EPA AQS monitor, we predicted monthly PM_2.5_ at the home addresses of the participants of each validation study for the time of the personal and ambient sampling. The pooled dataset of paired information on the surrogate exposures (spatio-temporal model prediction or nearest monitor) and the “true” exposure (personal PM_2.5_ of ambient origin) from the 5 cities was used to for the risk set calibrations.

### Potential confounders

Information on potential confounders is available every two years (every four years for diet information) from the follow-up questionnaires. Therefore, when appropriate, each woman was assigned updated covariate values for each questionnaire cycle. We examined possible confounding by numerous risk factors for all-cause mortality including: age (in months), race, physical activity, body mass index (BMI), hypercholesterolemia, and family history of MI. To control for smoking, we used lifetime smoking history to calculate pack-years (number of packs/day multiplied by number of years of cigarette smoking) and current smoking status (current/former/never). Diet was controlled for using a summary score based on the Alternate Healthy Eating Index (AHEI) [47]. As previously used in this cohort, the score included eight components of the AHEI: higher intakes of vegetables, fruit, nuts, soy and cereal fiber, alcohol consumption, high ratios of chicken plus fish to red meat and polyunsaturated to saturated fat, low intake of trans fat and multivitamin use of ≥5 years. To control for individual level socioeconomic status, we included several variables including nurses’ educational level, the occupation of both of the nurses’ parents when she was 16, marital status, and if applicable, husband’s education. To control for area-level socioeconomic status, we included area level information from the 2000 Census on tract level median income and house value for each residence. To control for long-term, regional, and seasonal patterns in mortality and pollution, we also adjusted all models for calendar year, season, and Census region.

### Statistical methods

Time-varying Cox proportional hazards models were used to assess the association of exposure to PM_2.5_ in the previous 12-months from either the spatio-temporal models or the nearest monitor with the incidence of all-cause mortality. Person-months of follow-up were calculated from June 2000 until the earliest of end of follow-up (May 2006), death, or loss to follow-up. All models were stratified by age in months and calendar month and year and were used to estimate hazard ratios (HRs) and 95% confidence intervals (CIs). Multivariate models were additionally adjusted *a priori* for the potential confounders listed above.

Risk set regression calibration (RRC) for time-varying exposures [22] was used to correct for bias due to exposure measurement error in the hazard ratios of all-cause mortality, utilizing the data from the external multi-city validation study. The goal of the measurement error correction was to quantify the difference in effect estimates induced by using ambient, as opposed to personal, measures of exposure to PM_2.5_ and to be able to apply these corrections in the setting of time-varying exposures. The “true” exposure of interest was assumed to be long-term personal PM_2.5_ of ambient origin, parameterized by the 12-month moving average. In brief, as an improvement over previous methods that applied the same calibration factor to all participants and time periods, the RRC method recalibrates the measurement error model for monthly PM_2.5_ exposure for each risk set observed in the main study by its counterpart in the validation study, and the 12-month average personal PM_2.5_ of ambient origin is then constructed from the monthly PM_2.5_ exposures estimated by the risk set-specific exposure measurement error models. A sandwich variance estimator is then used to calculate Wald-type asymptotic confidence intervals and *p*-values. Although adjusted for in the Cox model for all-cause mortality, calendar year was not included in the measurement error model because the validation studies were conducted over a 3 year calendar period. To account for the seasonal heterogeneity observed in the measurement error model for the spatio-temporal exposure predictions [30], we included an interaction term of season and PM_2.5_ to these measurement error models. Because the average number of people per household in a Census tract accounted for the between-city heterogeneity observed in the risk set calibration factors for the nearest EPA AQS monitor in the validation study [30], we included interaction terms of the number of people per household and PM_2.5_ in the measurement error models for this exposure. Non-linearity of all exposure response relationships was investigated through stepwise restricted cubic splines [48,49]. The analyses were performed in SAS version 9.2 and Fortran 90. User-friendly publicly available software to implement the RRC methodology is available for download [50]. To quantify the impact of measurement error adjustment, we calculated the percent difference in the HRs [((HR – HR _measurement error_)/HR)*100], as well as the percent increase in width in the confidence intervals [((UCL–LCL)-(UCL _measurement error_ –LCL _measurement error_))/(UCL–LCL)]*100], after adjustment.

## Results

Selected characteristics of the population over follow-up are presented in Table 1. The average age was 69.0 (SD 7.3), with a mean BMI of 25.8 (SD 7.4). Most (94%) of the women were Caucasian and 44% were never smokers. The average PM_2.5_ in the previous 12 months from the spatial temporal model was 12.0 μg/m^3^ (SD 2.8 μg/m^3^) and from the nearest monitoring location was 12.7 μg/m^3^ (SD 3.1 μg/m^3^).

**Table 1:** **Selected age-standardized characteristics of the Nurses’ Health Study participants throughout follow-up 6/2000-5/2006**

**Characteristic**	**Mean (SD) or %**
N^a^	108,767
Person-years^a^	628,186
Age (in years)^a^	69.0 (7.3)
Caucasian race	94
BMI (kg/m^2^)	25.8 (7.4)
Average PM_2.5_ over the previous 12 months	
Spatio-temporal model	12.0 (2.8)
Nearest USEPA monitor	12.7 (3.1)
Smoking status	
Never	44
Former	45
Current	10
Pack-years^b^	24.3 (21.8)
Physical activity (MET hr/week)	
<3	23
3 to <9	21
9 to <18	18
18 to <27	11
> = 27 MET	19
Alternative Healthy Eating Index	175 (95)
Hypertension	53
Hypercholesterolemia	61
Diabetes	11
Individual level SES	
RN degree	73
Housewife mother at age 16	64
Professional or manager father at age 16	26
Married	64
Husband’s education	
less than high school	4
high school	26
greater than high school	35
Census tract SES	
Median home value ($1,000)	170.2 (124.9)
Median income ($1,000)	63.4 (24.4)

^a^Value not age adjusted.

^b^Among ever smokers only.

Over 628,186 person-years of follow-up, there were a total of 8,617 non-accidental deaths. The associations for a 10 μg/m^3^ increase in spatio-temporal model predicted or nearest USEPA AQS monitor ambient average PM_2.5_ in the previous 12 months with all-cause mortality are shown in Table 2 with and without adjustment for measurement error. The age, calendar time, region and season adjusted HR was 1.20 (95%CI 1.11,1.29) for models using the spatio-temporal model predictions and 1.14 (95%CI: 1.06, 1.22) for models using the nearest USEPA monitor values. Both HRs remained elevated but became more comparable after adjustment for measurement error (1.27 (95%CI: 1.08, 1.48) and 1.26 (1.07, 1.48)), reflecting an increase of 5.8% for the spatio-temporal estimates, and 10.5% for the nearest monitor estimates. The effect estimates both remained statistically significant even with >100% widening of confidence intervals after accounting for the uncertainty due to exposure measurement error and the adjustment procedure.

**Table 2:** **Associations of 12-month average PM**_**2.5**_ **(per 10 μg/m** ^**3**^ **increase) from spatio-temporal model predictions or the nearest USEPA monitoring location values with all-cause mortality, with and without adjustment for exposure measurement error**

	**Spatio-temporal model**	**Nearest USEPA monitor**
Cases	8,617	8,617
Person-years	628,186	628,186
Basic HR (95%CI)^1^	1.20 (1.11, 1.29)	1.14 (1.06, 1.22)
Basic Measurement Error Adjusted HR (95%CI)^1,2^	1.27 (1.08, 1.48)	1.26 (1.07, 1.48)
% increase in HR^3^	5.8%	10.5%
% increase in 95% CIs^4^	122.2%	156.3%
Multivariable HR (95%CI)^5^	1.13 (1.05, 1.22)	1.12 (1.05, 1.21)
Multivariable Measurement Error Adjusted HR (95%CI)^2,5^	1.18 (1.02, 1.36)	1.22 (1.02, 1.45)
% increase in HR^3^	4.4%	8.9%
% increase in 95% CIs^4^	100.0%	168.8%

^1^Basic model: models stratified by age in months, adjusted for race, region, year and season.

^2^Additionally adjusted for exposure measurement error.

^3^[(HR – HR _measurement error_)/HR]*100.

^4^[((UCL–LCL)-(UCL _measurement error_ –LCL _measurement error_))/ (UCL–LCL)]*100.

^5^Multivariable: models stratified by age in months, adjusted for race. region, year, season, smoking status, pack-yrs, family history of MI, BMI, hypercholesterolemia, median family income in census tract of residence, median house value in census tract of residence, physical activity, race, Alternate Healthy Eating Index (AHEI), individual level socioeconomic status (nurses’ education level, occupation of both parents, marital status, and husband’s education).

Similar patterns were observed in multivariable models. Models unadjusted for measurement error were more comparable for the two exposure assignment methods (1.13 (95%CI: 1.05-1.22) for the spatio-temporal model predictions and 1.12 (95%CI: 1.05-1.21) for the nearest monitor estimates), and the magnitude of HR increases and increases in the width of the 95% confidence intervals were comparable to those from the basic models.

**Figure 1:**
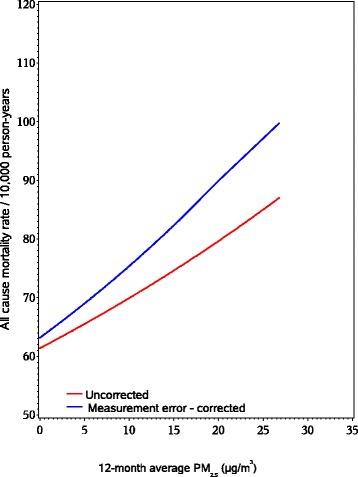
Measurement error corrected and uncorrected rates of all-cause mortality by 12-month spatio-temporal predicted average PM_2.5_. Multivariable models adjusted for age in months, adjusted for region, year, season, smoking status, pack-yrs, family history of MI, BMI, hypercholesterolemia, diabetes, hypertension, median family income in census tract of residence, median house value in census tract of residence, physical activity, race, Alternate Healthy Eating Index (AHEI), individual level socioeconomic status (nurses’ education level, occupation of both parents, marital status, and husband’s education).

**Figure 2:**
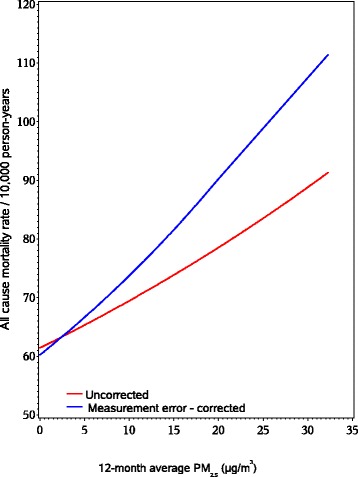
Measurement error corrected and uncorrected rates of all-cause mortality by 12-month nearest USEPA monitor average PM_2.5_. Multivariable models adjusted for age in months, adjusted for region, year, season, smoking status, pack-yrs, family history of MI, BMI, hypercholesterolemia, diabetes, hypertension, median family income in census tract of residence, median house value in census tract of residence, physical activity, race, Alternate Healthy Eating Index (AHEI), individual level socioeconomic status (nurses’ education level, occupation of both parents, marital status, and husband’s education).

As shown in Figure 1 (spatio-temporal model) and Figure 2 (nearest monitor), the multivariable adjusted predicted mortality rates for a given 12-month average PM_2.5_ level were higher when using the measurement error adjusted estimates, compared to estimates ignoring measurement error.

## Discussion

In analyses of this nationwide cohort of middle-aged and older women, increasing exposure to PM_2.5_ was associated with an increased risk of mortality. Contrary to our *a priori* hypothesis of elevated and more precise results using the spatio-temporal model predictions, the results were similar regardless of the method chosen for exposure prediction. Specifically, the HRs for 2000–2006 were 1.14 (95%CI: 1.05-1.23) and 1.13 (95%CI: 1.05-1.21) for predictions from the spatio-temporal prediction models and nearest USEPA monitoring location, respectively. Adjustment for measurement error increased the magnitude of the HRs by 10-15%, and also widened the confidence intervals, suggesting the presence of both classical and Berkson errors when using ambient, as opposed to personal exposures of ambient origin, as the exposures of interest [17].

Our estimated effects are lower than our previous findings in this cohort. Among women living in a selection of Northeastern and Midwestern states of the US between 1992–2002, each 10 μg/m^3^ increase in PM_2.5_ was associated with an HR = 1.26 (95%CI: 1.02-1.54) [51]. These differences are likely due to a combination of factors including different follow-up periods, the expansion to all contiguous states, differences in inclusion and exclusion criteria, and differences in parameterizations of the statistical models. However, our current results for the whole country 2000–2006 are similar to the equivalent meta-estimate for a 10 μg/m^3^ increase in PM_2.5_ from 19 cohorts participating in the European Study of Cohorts for Air Pollution Effects (ESCAPE) project (HR = 1.14; 95%CI: 1.04-1.26) [52].

Our measurement error correction method was designed to correct for the most likely source of error in long-term pollution studies, namely differences between ambient and personal levels of PM_2.5_ of ambient origin. Importantly, we were able to use these methods to correct for measurement error using both concentrations at the nearest monitoring location and predictions at the home address from spatio-temporal models. This allowed us to examine the impact of both methods of exposure prediction and the impact of measurement error on our findings. Notably, although additional information was needed to estimate the impact of measurement error from the nearest monitor, our results suggest similar levels of risk from the two prediction methods. An important point is that the measurements from the validation studies are also subject to measurement error. As long as these errors are uncorrelated with the errors in nearest monitor and spatio-temporal predictions, which we believe is likely to be true, the measurement error-corrected results will give valid point estimates and confidence intervals [60,61].

A number of methods to assess and quantify exposure measurement error have recently been proposed. In a study based in the Netherlands Cohort Study on Diet and Cancer, we recently used related non-time varying measurement error correction methods to assess the impact of measurement error on associations of air pollution and traffic parameters on lung cancer risk. Adjustment for measurement error to account for the differences between personal and ambient exposures led to modest increases in the HRs (0–3.3%) for exposures to black smoke and PM_2.5_ (9.7-37.2%), accompanied by substantial widening of the 95%CIs (10.2-216.8%) [53]. Other methods, including the use imputation based on random effects meta-analysis of correlations between personal and ambient exposures reported in the literature [54,55], have also been used to demonstrate increases in effect estimates and widening of 95%CIs in a study of heart rate variability [56]. Another group of methods have been developed to address errors induced by spatial modeling of exposures or spatial autocorrelation and have shown promise in simulation studies [17,57-59]. Overall, these methods suggest that air pollution studies are subject to complex measurement error structures, and that it is likely a variety of methods are needed to appropriately adjust the large number of study types.

There are a number of limitations to our study to note. First, although we were able to obtain information from a number of exposure panel studies, only a limited number (n = 5) of validation studies had information available to determine levels of PM_2.5_ of ambient origin. It is possible that differences in time-activity patterns between the participants in these validation studies and those in our study population could have been different, even with our approach of matching risk sets based on current age. There was also limited geographic coverage of the validation studies, which prevented us from applying region-specific adjustments. These differences in activity patterns and possible regional differences may cause our measurement error correction to incorporate error either away from or towards the null. Since most of the covariates adjusted for in the multivariable Cox models shown in Table 2 were not available in the validation study, we had to assume they are not the confounders in the measurement error model. Additionally, individuals in the validation studies wore personal sampling devices for short periods of time (median duration: 7 days), which were then used to calculate monthly “gold standard” exposures. The methods used here assume that the relationship between the personal measurements and the surrogate exposures observed on these days was representative of that would have been observed over an entire month. This is reasonable, given that the days within a month were chosen at random and we have shown in our validation study that the number of days each person participated had little impact on the size of the calibration factors [30]. We are also assuming that the month-specific relationships between the short-term personal and surrogate exposures collected in 1999–2002 can be used to correct 12-month average exposures between 2000 and 2006. The interclass correlation of 1-month exposures from the spatio-temporal model for all participants was 0.41 and the correlation between the 1-month and 12-month averages was 0.68; therefore, this is a reasonable assumption.

This study also has several major strengths. Our long follow-up period and consideration of residential address history allowed us to estimate time-varying exposures to PM_2.5_ over almost two decades. The availability of a wealth of follow-up data also allowed us to tightly control for a number of potential confounders in a time-varying manner, lessening concerns about residual confounding. The large number of cases allowed us to examine differences in regional patterns in risk and to examine risks after the availability of PM_2.5_ monitoring in the US. Lastly, our quantification of the potential bias due to measurement error induced by use of ambient levels of PM_2.5_ instead of personal exposures of ambient origin provides a sense of the potential level of underestimation in previous studies that may exist if similar associations between personal PM_2.5_ of ambient origin and spatio-temporal or nearest monitor predictions can be assumed.

## Conclusions

In this large nationwide cohort of middle-aged and elderly women, exposures to PM_2.5_ were associated with an increased risk of all-cause mortality using two complementary approaches to exposure assessment. There was evidence that this risk varied by region of the county even after adjustment for a number of lifestyle and demographic factors. Therefore, our study provides evidence that 12-month average PM_2.5_ exposures below the current USEPA standard are associated with an increased risk of all-cause mortality and that measurement error corrections should be implemented in studies whenever possible.

## Abbreviations

AHEI: Alternate healthy eating index
AQS: Air quality system
CI: Confidence interval
ESCAPE: European Study of Cohorts for Air Pollution Effects
GIS: Geographic Information System
HR: Hazard ratio
NHS: Nurses’ Health Study
PM_2.5_: Particulate matter less than 2.5 microns in aerodynamic diameter
PM_10_: Particulate matter less than 10 microns in aerodynamic diameter
RRC: Risk set regression calibration
USEPA: United States Environmental Protection Agency
